# Identification of conserved gene clusters in multiple genomes based on synteny and homology

**DOI:** 10.1186/1471-2105-12-S9-S18

**Published:** 2011-10-05

**Authors:** Anasua Sarkar, Hayssam Soueidan, Macha Nikolski

**Affiliations:** 1 LaBRI, CNRS/Université Bordeaux 1, 351 crs Libération, 33405 Talence, France; 2 Netherlands Cancer Institute, Plesmanlaan 121, 1066 CX Amsterdam, The Netherlands

## Abstract

**Background:**

Uncovering the relationship between the conserved chromosomal segments and the functional relatedness of elements within these segments is an important question in computational genomics. We build upon the series of works on *gene teams* and *homology teams.*

**Results:**

Our primary contribution is a local sliding-window SYNS (SYNtenic teamS) algorithm that refines an existing family structure into orthologous sub-families by analyzing the neighborhoods around the members of a given family with a locally sliding window. The neighborhood analysis is done by computing conserved gene clusters. We evaluate our algorithm on the existing homologous families from the Genolevures database over five genomes of the Hemyascomycete phylum.

**Conclusions:**

The result is an efficient algorithm that works on multiple genomes, considers paralogous copies of genes and is able to uncover orthologous clusters even in distant genomes. Resulting orthologous clusters are comparable to those obtained by manual curation.

## Background

Uncovering the relationship between the conserved chromosomal segments and the functional relatedness of elements within these segments is an important question in computational genomics. It is often suggested that regions with similar gene content among different species are evidence for phylogenetic relationship and trace through evolution the inheritance of function from a common ancestor. Within one genome, the presence of large duplicated blocks may be due to the ancient large-scale or whole genome duplication, while presence of segments with homologous genes, named *conserved gene clusters* in multiple genomes more likely indicates an evolutionary constraint for a functionally related group. Our primary contribution is a local sliding-window algorithm that starts from an existing protein family classification and produces two results: first, concerved gene clusters, and second, a subdivision of families into orhtologous subgroups. Our approach can be seen as using conserved gene clusters in order to sift through the family structure to uncover orthology. We evaluate the biological relevance of our approach on the example of Protoploid yeasts [1].

A number of studies indicate that regions of conserved homology among multiple species may result from functional pressure to keep these genes close, but it may also be conserved because the genomes under study have not sufficiently diverged. For the former, the most well known examples are that of operons in prokaryotes [2], but also the existence of functional interactions [3] and similar expression patterns [4] in closely located genes. For the latter, existence of conserved gene clusters is the computational basis for ancestral genome reconstruction [5] and search for ancestral homologs among genes in the same family [6]. Orthologs are homologous genes related by speciation [7,8] which retain the same functionality as their common ancestors. Homologous genes related by duplication within one lineage are called paralogs and generally differ in functionality [9-12]. A number of papers introduce algorithms to compute conserved gene clusters and orthologous groups, see for example, [13-16]. These approaches vary on a number of parameters. First, there are authors who consider strictly conserved chromosomal segments with similar gene order and orientation [17-19]. Second, come the approaches where one considers conserved contiguous regions but without co-linearity [13,20]. Third, the authors relax the definition of conserved regions by allowing gaps [18,21-24]. Four, paralogous gene copies within one chromosome are allowed in order to explore many-to-many homologous relationships [13,25]. Finally, some authors study the effect of varying the gap between adjacent neighbors [24,26,27].

In this paper we start from the notion of *gene teams* introduced in [28]. This model allows only one copy of a gene on a given chromosome. We relax this restriction by following the approach of *homology teams* defined in [13]. Furthermore, we set the gap threshold not only for adjacent genes, but by requiring the distance for any two genes considered as being neighbors to be smaller than a certain threshold. A similar choice was made in [20]. We call the obtained gene clusters *synteny teams.*

Our SYNS (SYNtenic teamS) algorithm refines existing families into orthologous sub-families, by analyzing the neighborhoods around the members of a given family with a locally sliding window. This is done for all pairs of chromosomes in multiple genomes on which family members appear. The pairwise conserved contiguous segments are agglomerated by relying on a partial homology and biological criteria introduced in [1] between segments. This results in larger conserved segments that we call *syntenic zones.* We evaluate our algorithm on the existing homologous families for five genomes of the Hemyascomycete yeasts from the Genolevures database [29]. Indeed, there already exists a sub-classification of these families into orthologous sub-families [1] that has undergone expert validation and thus can be used as a reference point for the evaluation of biological relevance of our results. We further illustrate the results of our method for the particular case of the Pdrp (pleiotropic drug resistance proteins) phylogenetic subfamily of ABC transporters that has been manually analyzed in [6].

## Methods

### 

In this section we define the notion of unordered conserved gene clusters that allows for paralogous copies and gaps on multiple genomes. Following the work of [20,30,31], we allow one homologous gene to appear more than once in one chromosome. We refine the approach of homology teams [13] by distinguishing between orthologous and paralogous copies of genes. Large syntenic zones are built my merging clusters based on genes common among them instead of directly merging the ordered chains with overlapping families as in [32]. For mathematical notations and examples in a textual format we follow [28].

**Definition 1*** A* chromosome *is defined as a pair c* = (Σ, *G*), *where* Σ = {*f*_1_, *f*_2_, …, *f_m_*} *is the set of homologous families and G* = (*g*_1_, *g*_2_, ..., *g_n_*) *is an ordered sequence of genes. Each* gene g*_i_* ∈ *G is a couple* (*p_i_*, *f_i_*), *where p_i_ is the position of gene g_i_on c and g_i_belongs to some homologous group f_i_* ∈ Σ*.*

Here, Σ is the alphabet for any chromosome *c* and *p_i_* is an integer. When it is necessary to indicate to which chromosome belongs a given gene, this is done by a subscript: (*p_i_*, *f_i_*)*_c_.*

**Definition 2 ***Given a chromosome c*, *with two genes g_i_* = (*p_i_*, *f_i_*) *and g_j_* = (*p_j_*, *f_j_*), *the* distance *between g_i_ and g_j_ is defined by* Δ(*g_i_*, *g_j_*) = |*p_i_– p_j_*|*.*

**Example 1*** Let c*_1_*and c*_2_*be two chromosomes over the same alphabet* Σ = {*f*_1_, *f*_2_, *f*_3_, *f*_4_} *of homologous families with genes on c*_1_*being* (1, *f*_2_), (2, *f*_1_), (4, *f*_4_), (7, *f*_3_), (8, *f*_1_), *and on c*_2_*being* (1, *f*_1_), (2, *f*_2_), (3, *f*_2_), (4, *f*_3_), (6, *f*_4_)*. This is denoted by:*

*c*_1_ = 〈*f*_2_*f*_1_*f_4_**f_3_*f*_1_〉,

*c*_2_ = 〈*f*_1_*f*_2_*f*_2_*f*_3_*f_4_〉.

Asterisks stand for genes that are unassigned to homologous groups; notice that * is not part of the alphabet Σ.

A gene subset *G′* ⊆ *G* induces the subset of families Σ′ denoted by Σ(*G*′) such that *f_i_* ∈ Σ′ if and only if there exists *g_i_* ∈ *G*′ such that *g_i_* = (*p_i_*, *f_i_*). A set of genes *G*′ from the same chromosome, forms a *chromosomal segment s* = (Σ′, *G*′, *c*) with or without gaps. When it is clear from the context, we will assimilate a set of genes *G*′ with the corresponding chromosomal segment.

For example, in the case of *G*′ = {(2, *f*_1_), (4, *f*_4_), (8, *f*_1_)} and alphabet Σ′ = Σ(*G*′) = {*f*_1_, *f*_4_}, *G*′ defines a chromosomal segment with gaps on *c*_1_ = 〈*f*_2_*f*_1_ * *f*_4_ * *f_3_*f*_1_〉. This segment *G*′ is non-contiguous on *c*_1_; the gaps correspond to (5, *), (6, *) and (7, *f*_3_).

**Definition 3 ***A chromosomal segment s* = (Σ′, *G*′, *c*) *is* contiguous *if for any two genes g_i_* = (*p_i_*, *f_i_*) *and g_j_* = (*P_i_*, *f_j_*) *from G*′ *and any psuch that p_i_* <*p* <*p_j_*, *either the gene g* = (*p*, *f*) *at the position p belongs to G' or this position corresponds to an asterisk. Otherwise*, *the segment is said to be* non-contiguous For example, *G*′ = {(4, *f*_4_), (7, *f*_3_), (8, *f*_1_)} on *c*_1_ = 〈*f*_2_*f*_1_ * *f*_4_ * *f_3_*f*_1_〉 forms a contiguous segment.

#### Synteny teams

Two genes *g_i_* = (*p_i_*, *f_i_*) and *g_j_* = (*p_j_*, *f_j_*) on the same chromosome are considered to be *neighbors* when Δ(*g_i_– g_j_*) <*δ* for a given threshold *δ* > 0. For a gene *g_i_*, we denote the set of neighbor genes *N_i_* to be centered around it, that is *N_i_* = {*g_k_* = (*p_k_*, *f_k_*) | *p_i_* – ⌊*δ/2*⌋ ≤ *p_k_* ≤ *p_i_* + ⌊*δ*/2⌋}.

**Definition 4*** A chromosomal segment s is called a δ—*segment *if every pair of genes of s is separated by a distance smaller than δ*, *that is s* = {*g_i_* | ∀*g_j_* ∈ *s*, Δ(*g_i_*, *g_j_*) <*δ*}*. A* window *w is a contiguous δ-segment.*

**Definition 5 ***We say that* Σ′ ⊆ Σ *is a δ—*subset *if there exists at least one δ—segment s*′ = (Σ′, *G*′, *c*) *such that* Σ*'* = Σ(*G'*)*. We say that s' is the* witness *of this δ—subset.*

**Example 2*** For δ* = 3, *the δ—subsets on chromosome c*_2_ = 〈*f*_1_*f*_2_*f*_2_*f*_3_ * *f*_4_〉 *are the following:*

*-* {*f*_1_, *f*_2_} *as witnessed by* ((1, *f*_1_), (2, *f*_2_)), ((1, *f*_1_), (3, *f*_2_)), *and* ((1, *f*_1_), (2, *f*_2_), (3, *f*_2_))*;*

*-* {*f*_2_, *f*_3_} *as witnessed by* ((2, *f*_2_), (4, *f*_3_)), ((3, *f*_2_), (4, *f*_3_)), *and* ((2, *f*_2_), (3, *f*_2_), (4, *f*_3_))*;*

*-* {*f*_3_, *f*_4_} *as witnessed by* ((4, *f*_3_), (6, *f*_4_))*.*

**Definition 6*** Let* Σ *be the set of homologous families over a set of chromosomes C. We say that* Σ′ ⊆ Σ *is a δ—*cluster *if* Σ′ *is a δ—subset for all chromosomes in some C*′ ⊆ *C*, *where |C*′| ≥ 2*. We say that the set of genes*

*witnesses the δ—cluster* Σ′*.*

A witness *S* is thus a set of all genes that participate in the segments witnessing the relevant (*δ*-subsets. Let Σ and Σ*'* to be two (*δ*-clusters such that Σ ∩ Σ′ ≠ *∅*. Let *S* and *S*′ be the corresponding witness sets. Denote by *S*_∩_ and  the sets of genes in each of these witness sets that are members of the families in Σ ∩ Σ′.

**Definition 7 ***A δ—cluster* Σ *is said to be a* (*δ*-synteny *if* (a) *the corresponding witness set S has genes belonging to at least two different chromosomes and* (b) *there does not exist a δ-cluster* Σ′ *with a witness set S*′ *such that*

**Figure 1:**
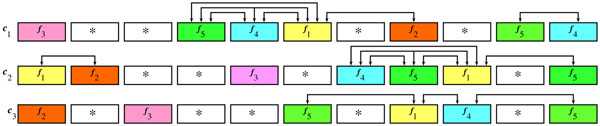
**Example of *δ*-clusters and *δ*-syntenies** The non-trivial δ—syntenies for the example 3 that cover *c*_1_, *c*_2_ and *c*_3_ are {*f*_4_, *f*_5_}, {*f*_4_, *f*_1_} and {*f*_5_, *f*_1_}*;* that cover *c*_1_ and *c*_2_ are {*f*_1_, *f*_2_} and {*f*_4_, *f*_5_, *f*_1_}*.* Colors indicate homology relationships. Connections indicate the relevant δ—clusters.

**Example 3*** Let c*_1_, *c*_2_*and c*_3_*be chromosomes as shown in figure*1.

*c*_1_ = 〈*f*_3_ * *f_5_*f*_4_*f*_1_ * *f*_2_* f_5_*f*_4_〉

*c*_2_ = 〈*f*_1_*f*_2_ * *f_3_*f_4_*f*_5_*f*_1_ * *f*_5_〉

*c*_3_ = 〈*f*_2_ * *f*_3_ **f_5_*f_1_*f*_4_ * *f*_5_〉

*Let* (*δ* = 3. *We obtain the following non-trivial δ—clusters:* {*f*_4_, *f*_1_}, {*f*_5_, *f*_1_}, {*f*_4_, *f*_5_, *f*_1_}, {*f*_1_, *f*_2_} *and* {*f*_4_, *f*_5_} *between c*_1_*and c*_2_*; and* {*f*_1_, *f*_5_}, {*f*_1_, *f*_4_} *and* {*f*_4_, *f*_5_} *between c*_1_*and c*_3_*. The non-trivial δ-syntenies are* {*f*_4_, *f*_5_}, {*f*_1_, *f*_2_}, {*f*_4_, *f*_1_}, {*f*_5_, *f*_1_} *and* {*f*_4_, *f*_5_, *f*_1_}*.*

The superset inclusion in definition 7 implies that for the computational purposes there is no need to consider the smaller of the two sets and thus causes *merging* of the syntenies if the witness of one synteny is a complete subset of another in our algorithm.

**Figure 2:**
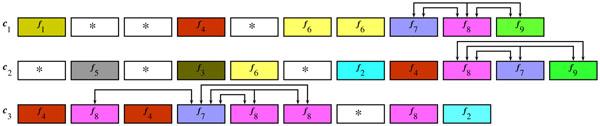
**Merging of *δ*-syntenies** Merging among *δ*-syntenies for chromosomes in the example 4: when considering *f*_8_, *δ*-cluster {*f*_8_, *f*_9_} is merged in the <5-synteny {*f*_7_, *f*_8_, *f*_9_}. Colors indicate the homology relationship. Connections indicate the relevant *δ*—clusters (only those relevant for merging are shown).

**Example 4*** Let c*_1_, *c*_2_*and c*_3_*be three chromosomes in figure*2*.*

*c*_1_ = 〈*f*_1_ * *f_4_* f_6_*f*_6_*f*_7_*f*_8_*f*_9_〉

*c*_2_ = 〈*f_5_* f_3_*f*_6_* f_2_*f*_4_*f*_8_*f*_7_*f*_9_〉

*c*_3_ = 〈*f*_4_*f*_8_*f*_4_*f*_7_*f*_8_*f*_8_* f_8_*f*_2_〉

*Let* (*δ* = 3 *and consider f*_8_*. Non-trivial δ—clusters are:* {*f*_7_, *f*_8_, *f*_9_}, {*f*_7_, *f*_8_} *and* {*f*_8_, *f*_9_} *between c*_1_*and c*_2_, {*f*_7_, *f*_8_} *between c*_1_*and c*_3_*and* { *f*_8_, *f*_2_}, {*f*_7_, *f*_8_} *and* {*f*_8_, *f*_4_} *between c*_2_*and c*_3_*. Therefore*, *we obtain the following non-trivial δ—syntenies:* {*f*_7_, *f*_8_, *f*_9_}, {*f*_7_, *f*_8_}, {*f*_8_, *f*_2_} *and* {*f*_8_, *f*_4_}*. Notice that the δ-cluster* {*f*_7_, *f*_8_, *f*_9_} *covers witnesses of the δ-cluster* {*f*_8_, *f*_9_}, *but the witnesses of the δ-cluster* {*f*_7_, *f*_8_} *on chromosome c*_3_*do not witness the δ-cluster* {*f*_7_, *f*_8_, *f*_9_}*. Therefore*, *we merge the δ-cluster* {*f*_8_, *f*_9_} *in th e δ-synteny* {*f*_7_, *f*_8_, *f*_9_}*; however*, {*f*_7_, *f*_8_} *remains as a separate δ-synteny.*

We have seen that a (*δ*—synteny must contain the maximal (*δ*—cluster with respect to subset inclusion. All (*δ*—syntenies for a set of chromosomes *C*, with |*C*| >= 2 are included in the result. Such a synteny set is informally called a *synteny team* following the terminology introduced in [28,32] for gene teams.

**Definition 8*** Given a δ—synteny team**we say that* Σ*_i_and* Σ*_j_are transitively connected if the witnesses S_i_ and S_j_ overlap*, *that is* |*S_i_* ∩ *S_j_*| ≥ 1*. We further define a δ*-zone *as a union of transitively connected δ-syntenies* Σ*_i_and* Σ*_j_.*

**Figure 3:**
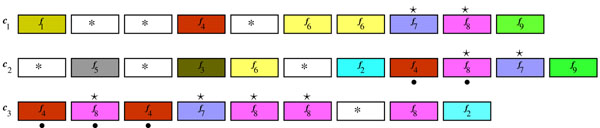
**Agglomeration of *δ*-syntenies in *δ*-zones** Example of *δ*-syntenies’ agglomeration by transitivity (see example 5): *δ*-clusters Σ_1_ = {*f*_7_, *f*_8_} and Σ_2_ = {*f*_4_, *f*_8_} are witnessed by gene sets indicated by ✰ and • symbols, respectively. They are merged in one *δ*—zone {*f*_4_, *f*_8_, *f*_7_} based on witness intersection {(9, *f*_8_)_*c*_2__, (2, *f*_8_)_*c*_3__}*.*

**Example 5*** Consider C* = {*c*_1_, *c*_2_, *c*_3_} *from example 4 and δ* = 3*. Suppose that we compute clusters in the neighborhood of f*_8_*. Non-trivial syntenies are the following:* Σ_1_ = {*f*_7_, *f*_8_} *for witness S*_1_ = {(8, *f*_7_)_*c*_1__, (9, *f*_8_)_*c*_1__, (9, *f*_8_)_c_*2*__, (10, *f*_7_)_*c*_2__, (2, *f*_8_)_*c*_3__ (4, *f*_7_)_*c*_3__, (5, *f*_8_)_*c*_3__, (6, *f*_8_)_*c*_3__} *and* Σ_2_ = {*f*_4_, f_8_} *for witness S*_2_ = {(8, *f*_4_)_*c*_2__, (9, *f*_8_)_*c*_2__, (1, *f*_4_)_*c*_3__, (3, *f*_4_)_*c*_3__, (2, *f*_8_)_*c*_3__}*. Notice*, *that* Σ_1_ ∩ Σ_2_ = {*f*_8_} ≠ *∅and S*_1_ ∩ *S*_2_ = {(9, *c*_2_, *f_8_*), (2, c_3_, *f*_8_)} ≠ *∅. We obtain one non-trivial δ—zone* {*f*_4_, *f*_8_, *f*_7_} *by agglomerating δ—syntenies* Σ_1_*and* Σ_2_*based on the transitivity* (*see figure*3)*. Notice that this leaves the gene* (8, *f*_8_)_*c*_3__, *out of the δ-zone.* The transitivity relationship in the SYNS algorithm combines each pair of two *δ—*syntenies sharing at least one witness into one *δ*-zone. The notion of a *δ—*zone aims at uncovering even distant evolutionary relationships based on conservation of gene content within neighborhoods. It is slightly amended based on the following two considerations.

1. Several paralogous genes may exist on the same chromosome. When two or more paralogs appear within one window of size *δ*, we include them in the same witness set of a *δ*-synteny since it is not possible to computationally distinguish between them.

2. It may happen that two distinct *δ*-syntenies share only one paralogous gene. This is what we call a *weak bond.* Creating a *δ*-zone based on a single gene intersection may either lead to a *δ*-zone that is phylogenetically valid or may create an erroneous result (see [6]).

**Definition 9*** Given a δ—synteny team**and its witness set S* = {*S_i_*, *S_j_*} *we say that S forms a* weak bond *if* |*S_i_* ∩ *S_j_*| = 1*. We further define g* = *S_i_* ∩ *S_j_ to be the* witness of a weak bond*.*

The *δ—*zone {Σ*_i_*, Σ*_j_*} resulting from a weak bond may be erroneous. We rely on phylogeny to solve this issue. We consider a total order over all the species under study defined by phylogeny: *a* ≺ *b* if species *b* has diverged from the common ancestor earlier than species *a* (≺ corresponds then to the relative speciation time). When no other witness from *a* other than *g* exists, we split the erroneously obtained synteny in two parts: one that contains the orthology relationships within a given family *f* and another one that keeps the supposed paralogs. The details of how this is done are presented in Results section.

**Definition 10*** Let**be a δ— synteny team over the witness set S* = {*S_i_*, *S_j_*} *such that* |*S_i_*| > |*S_j_*| *and let g* = *S_i_* ∩ *S_j_ be the witness of a weak bond. If g is from the biggest species according to* ≺ *in S_j_*, *we say that S_i_witnesses a maximal* orthologous (*δ*-synteny Σ*_i_and S'_j_* = *S_j_* \ *g witnesses a* paralogous *δ*-synteny Σ*_j_.*

**Figure 4:**
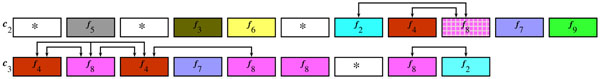
**Example of a weak bond** Example of a weak bond among *δ*-syntenies considering family *f*_8_*:* {(9, *f*_8_)_*c*_2__} is the witness of a weak bond between the *δ*-syntenies {*f*_8_, *f*_2_} and {*f*_4_, *f*_8_}*.* Colors indicate the homology relationship. Connections indicate the relevant *δ*—clusters. Crossed box indicates the witness of a weak bond.

**Example 6*** Consider C′* = {*c*_2_, *c*_3_} *from example 4 and figure*4*supposing that c*_3_ ≺ c_2_*and consider neighborhoods around f*_8_*with* (*δ* = 3*. Two non-trivial δ-syntenies are connected by a weak bond:* Σ_1_ = {*f*_8_, *f*_2_} *with witness S*_1_ = {(8, *f*_2_)_*c*_2__, (9, *f*_8_)_*c*_2__, (8, *f*_8_)_*c*_3__, (9, *f*_2_)_*c*_3__} *and* Σ_2_ = { *f*_4_, *f*_8_} *with witness S*_2_ = {(7, *f*_4_)_*c*_2__, (9, *f*_8_)_*c*_2__, (1, *f*_4_)_*c*_3__, (2, *f*_8_)_*c*_3__ (3, *f*_4_)_*c*_3__, (5, *f*_8_)_*c*_3__}*. Indeed*, {(9, *f*_8_)_*c*_2__} *is the witness of this weak bond. Since c*_3_ ≺ *c*_2_, *then* Σ_2_*is the maximal orthologous δ-synteny with witness S*_2_, *while* Σ_1_*is the one with the paralogous copy of f*_8_ (*at position 9 on c*_2_)*. The set S*_1_*becomes* Members of a family are split into an orthologous and paralogous subsets present in different syntenies. At the end of our procedure, only the largest orthologous (*δ*-zone and the non-intersecting paralogous (*δ*-zones covering any given homologous family remain in the result.

### Syntenic TeamS algorithm

**Figure 5:**
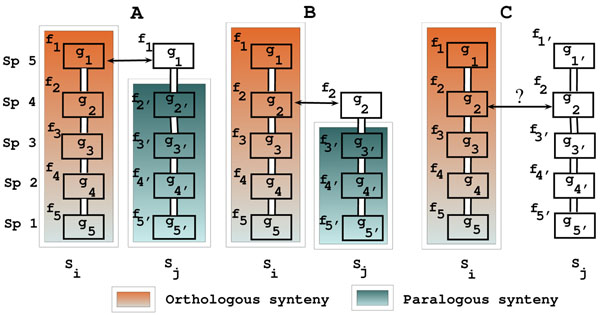
**Three topologies for a weak bond** Examples of topologies where two *δ*-syntenies Σ*_i_* and Σ*_j_* with witness sets *S_i_* and *S_j_* have a weak bond. Species are ordered by phylogenetic order *Sp_i_* ≺ *Sp_i_*_+1_*.* Cases A (*g*_1_ is the witness of weak bond) and B (*g*_2_ is the witness of weak bond) are considered to be plausible from the evolutionary perspective, while case C (*g*_2_ is the witness of weak bond) is difficult to explain. Different colors represent the orthologous and paralogous *δ*-syntenies emerging from these cases. Vertical links represent synteny while horizontal arrows represent the weak bond.

In this section, we present the SYntenic TeamS (SYNS) algorithm which computes *δ—*zones in multiple genomes. In previous work gene teams between two chromosomes of size m and *n* are computed by an *O*(*m* + *n*)*log^2^*(*m* + *n*) algorithm consisdering only one-to-one homologous relationships [32]. The approach by [20] solves the ordered gene clusters problem by proposing a directed acyclic graph model and an NP-hard longest path solution; results contain maximal but also non-maximal orthologous clusters. Our approach relies on the same sliding-window general approach as in [20]. However, we gain in time efficiency by limiting the sliding of the window only around positions of family members. Given a set of families Σ and a predefined window size *δ*, we examine neighborhoods of each family *f* ∈ Σ in all chromosomes. For all genes of *f* including paralogous copies, we consider a neighborhood from –*δ* to +*δ* around them. This neighborhood is examined by a sliding window of size *δ* and we form sets of genes corresponding to families in a given window position. These sets are intersected to look for common gene content if they belong to different chromosomes. The intersections define synteny conservation within the family neighborhoods by using definitions in Methods section. We further look for transitivity among *δ—*syntenies and build (*δ*-zones. To do this, we search for overlaps among witnesses of *δ—*clusters. If the witness intersection size is > 1 then the *δ—* syntenies are agglomerated to form one *δ—*zone. Three different cases corresponding to phylogenetic topologies shown in figure 5 are considered for solving the weak bond problem. Let *S_i_* and *S_j_* to be the two witnesses connected by a weak bond, we sort the genes of these witnesses according to the ≺ order of speciation. If the witness of a weak bond occurs in the biggest species according to ≺ or if there is no any other witness from a bigger species, then we consider that (cases A and B in figure 5) the two clusters define a valid (*δ*-zone. Case C in figure 5 shows the situation where forming a (*δ*-zone can not be justified from the evolutionary perspective. For cases A and B we continue to search for paralogous gene clusters. We gather all maximal *δ—*zones in the final result.

#### Time complexity

Table 1 shows the comparative time complexity analysis of our approach and other existing ortholog detection algorithms for the cases where such information is available. In the SYNS algorithm, we consider that one homologous family *f* may appear in at most *c* × *t* locations in all genomes, where c is the total number of chromosomes and *t* is the maximal number of paralogous copies. Given that we explore neighborhoods of size 2 × *δ* + 1, the number of all windows of size *δ* for *f* is (*δ* + 1) × *c* × *t*. The computation of all witnesses for a given family takes *O*(((*δ* + 1) × *c* × *t*)^2^). If in this computation all the possible intersections are non-empty, then in the worst case scenario we obtain for *f* the set of (*δ*-clusters of size ((*δ* +1) × *c* × *t*)^2^. Which implies that the (*δ*-synteny computation takes *O*(((*δ* + 1) × *c* × *t*)^4^); which is repeated for all families *f* ∊ Σ.

**Table 1:** **Comparison of time complexity of OrthoMCL, GCFinder and SYNS** All experiments have been run on the dual-core Intel Xeon 2.33 GHz server. Results are also available for MultiParanoid (approx. 2 hours run time) and CoCo-CL (approx 3 hours run time) for which no time complexity is found in literature.

Method	Time complexity	Execution time	Notations Used
OrthoMCL	*O*(*Nk^2^*)	76min (excluding Blast)	*N* = #genes, *k* = pruning constant

GCFinder Ordered Unordered	*O*(*nd*(*k* + *d*)) *O*(*k^2^nD*(*tD* + 1)*^k^*^- 1^)	1546min interrupted after 3 days	*n* = #families *k* = #genes, *d* = window size *D* = max #genes in a window *t* = max # paralogous copies of a gene in a chr.

SYNS	*O*(*n*((*δ* + 1) × *c* × *t*)^4^)	15min	*n* = #families, c = #chr., *δ* = window size, *t* = max # of paralogous copies of a gene in a chr.

### Evaluation of results

The Genolevures database provides families of proteins across the phylum of hemyascomycetous yeasts. To evaluate the performance of our algorithm, we have executed it on the existing families from the Genolevures Release 3 Candiate 3 (2008-09-24) [33], [29] with 4949 families covering 25196 protein coding genes from five protoploid yeast species [1].

#### Comparison with other methods

The critical window-size parameter *δ* of SYNS was set to 7 for all experiments. This value was obtained in order to match our results with the previously defined and expert validated orthologous subgroups [1]. We have compared the orthologous groups obtained by SYNS on the yeast data to those obtained by the following methods: Coco-cl [34], MultiParanoid [35] and OrthoMCL [36]. Table 2 shows the numbers of orthologous groups classified by these methods. OrthoMCL [36] was run with default *inflation index*= 1.5, *e-value cut-off*= –5 and *percent match cut-off* = 50 values starting from input fasta files. Coco-cl was run recursively starting with fasta sequences with *boostrap threshold score*= 1 and *split score*= 0.5 and using ClustalW for multiple sequence alignment. Multiparanoid was run using default parameters (no cut-off and no duplicate appearance of gene in clusters), using BLOSUM62 matrix for Blast alignments. Table 2 shows the total number of classified proteins and the total number of orthologous groups detected by SYNS and these algorithms using the original Genolevures families as a baseline [33]. In comparison with the SONS method, the SYNS classifies a comparable number of proteins, but generates more orthologous groups, implying that these groups are more fine-grained.

We compare the orthologous groups between the SYNS method and those obtained by other algorithms in table 3. To compare two classifications we first look at how many groups are identical between two methods (Id column) and compute the similarity value (between 0 and 1) over the intersection of the covered protein sets (for definition see [33]). Second, we analyze the differences between two classifications. For these we report the number of proteins that are classified only by the SYNS (SYNS column) when compared to those only classified a given method (meth. column). The remaining differences are classified according to granularity: a split when a group obtained by a given method is split into multiple subgroups by the SYNS algorithm, a merge in the opposite case, and messy when the split/merge relationship is complicated. We further analyze the differences with respect to SONS classification case by case (available at http://www.cbib.u-bordeaux2.fr/redmine/projects/syns/files). We have found that in the case of splits between the resulting groups (50 groups in table 3, the more fine-grained groups obtained by the SYNS algorithm are more functionally relevant in general. For the cases of merges (141 groups) and messy events (70 groups) there is no clear-cut qualitative difference. However, for these 211 cases more functionally plausible groups can be obtained by SYNS when using a smaller window size *δ* = 5. Overall, SYNS method appears to be the best match with the curated SONS results [1], while relying on a clear mathematical definitions and having satisfactory running time.

**Table 2:** Comparisons of SYNS and other classifications with the existing family structure as baseline

Method	# proteins	Protein coverage	# groups
OrthoMCL	23399	92	4146
MultiParanoid	15937	63	15888
Coco-cl	24396	96	5252
GCFinder	10080	40	1779
SONS	24016	95	5424
SYNS	25147	99	6441
Genolevures Families	25196	100	4949

**Table 3:** **Comparison of different computations of orthologous clusters with SYNS results on the Genolevures data** Each line compares a given method with the SYNS; we report the number of genes classified only in the given method (meth), only by the SYNS algorithm (SYNS), the similarity value (sim) between two cluster sets (varying between 0 and 1 as defined in [33]), the number of genes that appear as singletons, the number of splits and merges between two cluster sets as well as the number of unclassifiable cases (messy).

Method	Id	sim	meth	SYNS	singls	merges	splits	messy
OrthoMCL	3447	0.76	41	1794	1044	594	18	32
MultiParanoid	4325	0.26	4	20518	1988	4	121	1
Coco-cl	3632	0.82	42	793	774	383	511	103
GCFinder	470	0.24	769	9781	3417	749	4	46
SONS	4968	0.90	27	1158	874	141	51	70

#### Analysis of two protein families

We illustrate the functional relevance the SYNS algorithm by considering the classification of Pdrp (pleiotropic drug resistance transporter proteins) subfamily performed in [6]. This is a subset of the PDR proteins from the GL3C0025 (total 60 proteins) Genolevures family. We compare this manual analysis with the results obtained automatically by SONS and SYNS algorithms.

Seven SONS, six SYNS and seven groups obtained by manual curation provide hypothethis on the evolution of this protein family. The manually curated orthologous groups are confirmed by gene cluster analysis. But in some cases the results differ. Groups *P*_1_ through *P*_4_ in table 4 denote four orthologous groups over five species annotated in [6] according to their *S. cerevisiae* members, namely Pdr12p group (*P*_3_, 5 members), Snq2p group (*P*_1_ + *P*_2_, 5+4=9 members) and Pdrp5p/15p group (*P*_4_, 3 members). Groups *P*_5_ through *P*_7_ in table 4 contain genes whose relationship to Pdr5p/15p is based on phylogenetic evidence only [6]. Three tandem gene repeats appear in ERGO (*Eremothecium gossypii*), KLLA (*Kluyveromyces lactis*) and SAKL (*Saccharomyces kluyveri*) and are found in a similar neighborhood [6] in groups *P*_1_ and *P*_2_.

Comparatively to the SONS classification, our approach proposes a more conservative classification for these proteins into orthologous groups. Indeed, SONS exclude ZYRO0D17710g from the Snq2/YNR070w phylogenetic cluster, while re-grouping the remaining proteins belonging to *P*_1_ and *P*_2_. Moreover, according to [6], SAKL0F04312g belongs to the Aus1p/Pdr11p group which has no shared neighborhood in pre-WGD five species. Thus, it is not surprising that this gene is missing in the SYNS classification (SONS algorithm classifies it in an independent group, not shown in table 4).

**Figure 6:**
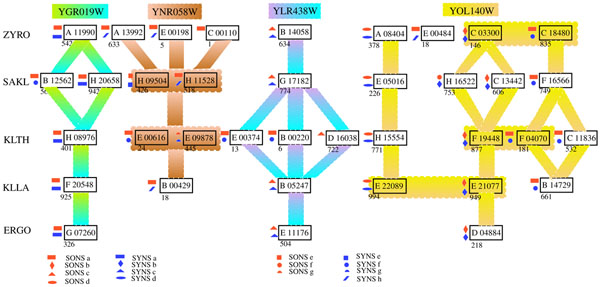
**Analysis of the Pdrp family** Relationships between the 57 members of GL3C0026 family based on their functional annotations. Each line lists genes from one species (indicated on the left); each box represents one gene. For example line ZYRO, first box on the left A11990 stands for ZYROA11990 gene. The numbers below the boxes represent the relative gene order (position) on the chromosomes. Genes with similar functional annotations are connected using the same color.

A similar analysis is done for the GL3C0026 family that has 57 members and four different functionally annotated groups. Figure 6 illustrates the evolutionary pattern based on the combination of phylogenetic analysis and functional annotations of this family. SONS algorithm produces 7 orthologous gene clusters, while SYNS generates 8 clusters functionally more relevant. Both SONS and SYNS successfully classify the L-ornithine transaminase (OTAse) group (with the *S. cerevisiae* member YLR438w CAR2). However, SONS classification fails to distinguish the YGR019w UGA1 Gamma-aminbutyrate (GABA) transaminase group from the YNR058w amino-pelargonic acid aminotransferase (DAPA) group. On the contrary, SYNS method separates the cluster having the YGR019w UGA1 gene according to its functional anotation. Our algorithm also succeeds to correctly distinguish the single orthog gene clusters from the YGR019w UGA1 group. For the YOL140w ARG8 Acetylornithine aminotransferase group, both SONS and SYNS algorithms provide similar conserved gene clusters. However, SONS erroneously mixes some genes of this group with YGR019w UGA1 cluster and YNR058w BIO3 cluster, whereas SYNS algorithm succeeds to distinguish them. The combined functional annotations and neighborhood analysis support the evolutionary pattern illustrated in figure 6 for the GL3C0026 family. Therefore we can conclude that the final *δ*-zones in our algorithm may preserve a functionally meaningful conserved gene clusters.

**Table 4:** **Comparisons of orthologous clusters subdividing the Pdrp Genolevures family** The Pdrp Genolevures family GL30025 as analysed by a) SONS results b) SYNS results c) after manual curation. The comparisons have been performed over the same sets of genes as in figure 3 in [6] for the Pdrp ”sensu stricto” proteins subset of the GL3C0025 family.

SONS orthologous groups	SYNS orthologous groups	Manual curation
*S*_1_= {ZYRO0A04114g SAKL0C11616g SAKL0C11704g KLTH0A01914g ERGO0B08140g ERGO0B08162g KLLA0D03432g KLLA0D03476g}	*Y*_1_= {ZYRO0A04114g SAKL0C11616g SAKL0C11704g KLTH0A01914g ERGO0B08140g ERGO0B08162g KLLA0D03432g KLLA0D03476g ZYRO0D17710g}	*P*_1_ = {ZYRO0A04114g SAKL0C11616g KLTH0A01914g ERGO0B08140g KLLA0D03432g}
*S*_2_= {ZYRO0D17710g}		*P*_2_ = {ZYRO0D17710g KLLA0D03476g ERGO0B08162g SAKL0C11704g}

*S*_3_ = {SAKL0C05654g SAKL0H10670g KLLA0B09702g ZYRO0F08866g ZYRO0F08888g}	*Y*_2_= {SAKL0C05654g SAKL0H10670g KLLA0B09702g ZYRO0F08866g ZYRO0F08888g}	*P*_3_ = {SAKL0C05654g SAKL0H10670g KLLA0B09702g ZYRO0F08866g ZYRO0F08888g}

*S*_4_ = {ZYRO0D11836g ZYRO0D11858g ZYRO0D11880g}	*Y*_3_ = {ZYRO0D11836g ZYRO0D11880g ZYRO0D11858g}	*P*_4_ = {ZYRO0D11836g ZYRO0D11880g ZYRO0D11858g}

*S*_5_ = {SAKL0G08008g KLLA0F21692g}	*Y*_4_ = {SAKL0G08008g KLLA0F21692g}	*P*_5_ = {SAKL0G08008g KLLA0F21692g}

*S*_6_= {ERGO0G05126g}	*Y*_5_ = {ERGO 0 G0 5 126g}	*P*_6_ = {ERGO0G05126g}

*S*_7_= {KLTH0G19448g KLTH0E17138g}	*Y*_6_ = {KLTH0G19448g KLTH0E17138g}	*P*_7_ = {KLTH0G19448g}

## Conclusion

The double goal of this study is to identify locally conserved gene clusters and to use them in order to subdivide an existing family structure into orthologous groups. To this end, we define a model for unordered local synteny and propose an algorithm to identify conserved gene clusters and their division into orthologous and paralogous clusters among multiple genomes. To validate our approach we have executed our method for the five Hemyascomycetous yeasts and genomes and examined the conserved non-overlapping gene clusters that arise from each homologous family of Genolevures database [29]. Our approach shows 99% protein coverage for existing homologous groups.

We perform similar comparisons with the existing SONS groups [6] over the Genolevures families. The 90% similarity between our approach and SONS groups indicates that our automatic method comes close to the manually curated results, especially since part of the differences between these groups can be explained by the non-classification of the paralogous conserved gene clusters by SONS. This confirms the pertinence of our definition of conserved neighborhoods based on transitivity and phylogenetic constraints that make it possible to include tandem repeats as well as loss, fusions or transpositions of gene copies in chromosomal rearrangements of genomes. The SYNS method makes it possible to distinguish between orthologous and paralogous conserved gene clusters and thus makes it possible to include tandem repeats as well as loss, fusions or transpositions of gene copies in chromosomal rearrangements of genomes. This implies that the proposed sliding window and partial traversal approach, efficiently produces biologically relevant conserved gene clusters and corresponding orthologous groups with *O*(*n*((*δ* + 1) × *c* × *t*)^4^) worst-case complexity, for a pre-defined window size *δ.*

## Competing interests

The authors declare that they have no competing interests.

## Authors' contributions

Conceived and designed the experiments: AS, MN. Performed the experiments and analyzed the data: AS. Wrote the paper: AS, HS, MN.
